# Retraction: Down-regulation of MRPS23 inhibits LPS-induced proliferation and invasion *via* regulation of the NF-κB signaling pathway in osteosarcoma cells

**DOI:** 10.1039/d3ra90071a

**Published:** 2023-08-03

**Authors:** Ai-Guo Liu, Ke-Lin Xu, Wei-Lin Wang, Bing-Kang Zhou, Qing-Gong Guo

**Affiliations:** a Department of Orthopaedics, The First Affiliated Hospital of Henan University Kaifeng 475000 China; b Department of Orthopaedics and Traumatology, Wuxi Hospital Affiliated to Nanjing University of Chinese Medicine No. 8 Zhongnan West Road Wuxi 214000 China xukelin_orth@163.com +86-510-88859999 +86-510-88859999

## Abstract

Retraction of ‘Down-regulation of MRPS23 inhibits LPS-induced proliferation and invasion *via* regulation of the NF-κB signaling pathway in osteosarcoma cells’ by Ai-Guo Liu *et al.*, *RSC Adv.*, 2019, **9**, 10561–10568, https://doi.org/10.1039/C8RA08973F.

The Royal Society of Chemistry hereby wholly retracts this *RSC Advances* article due to concerns with the reliability of the data.

Fig. 2D sh-MRPS23 panel is identical to Fig. 4C sh-MRPS23 panel (bottom left) and has some strong similarity with Fig. 4C sh-MRPS23 + LPS + PDTC panel (bottom right). A section in the panel for Fig. 2D sh-MRPS23 has strong similarity with Fig. 4C sh-Control + LPS + PDTC panel (top right). A section in the panel for Fig. 2D sh-control has strong similarity to a section in Fig. 4C sh-control + LPS + PDTC panel (top right).

In Fig. 4C a section in the panel for sh-control (top left) is a stretched version of a section in the panel sh-control with LPS and PDTC (top right).

Fig. 3C contains multiple duplicated images. The first four tumour images in the Control group are identical to the last four images in the sh-MRPS23 + LPS group. The third image in the control group is identical to the first image in the sh-control + LPS group. The final image in the control group is identical to the third image in the sh-MRPS23 group. The third image in the sh-MRPS23 group is identical to the first image in the sh-MRPS23 + LPS group.

The authors were asked to provide the raw data for this article but did not respond. Given the significance of the concerns about the validity of the data, and the lack of raw data, the findings presented in this paper are not reliable.

The authors were informed about the retraction of the article but have not responded.

Signed: Laura Fisher, Executive Editor, *RSC Advances*

Date: 18/7/2023

## Supplementary Material

